# A Template Method Leads to Precisely Synthesize SiO_2_@Fe_3_O_4_ Nanoparticles at the Hundred-Nanometer Scale

**DOI:** 10.3390/ma17174325

**Published:** 2024-08-31

**Authors:** Jinying Zhang, Xinye Wang, Jiaxing Yang, Yexiaotong Zhang

**Affiliations:** 1 Beijing Key Laboratory for Precision Optoelectronic Measurement Instrument and Technology, School of Optics and Photonics, Beijing Institute of Technology, Beijing 100081, China; 3120205353@bit.edu.cn (X.W.); 3120220654@bit.edu.cn (J.Y.); 3120220661@bit.edu.cn (Y.Z.); 2 Yangtze Delta Region Academy, Beijing Institute of Technology, Jiaxing 314001, China

**Keywords:** SiO_2_@Fe_3_O_4_, core-shell structured nanoparticles, template method, photonic crystals, controllable synthesis method

## Abstract

Constructing photonic crystals with core-shell structured nanoparticles is an important means for applications such as secure communication, anti-counterfeiting marking, and structural color camouflage. Nonetheless, the precise synthesis technology for core-shell structured nanoparticles at the hundred-nanometer scale faces significant challenges. This paper proposes a controlled synthesis method for core-shell structured nanoparticles using a template method. By using 100 nm diameter silica nanospheres as templates and coating them with a ferroferric oxide shell layer, SiO_2_@Fe_3_O_4_ core-shell structured nanoparticles with regular morphology and good uniformity can be obtained. The study experimentally investigated the effects of feed amount, modifiers, temperature, and feed order on the coating effect, systematically optimizing the preparation process. Centrifugal driving technology was used to achieve structural colors in the visible wavelength range. Additionally, the method successfully created well-defined and uniform core-shell structured nanoparticles using 200 nm diameter silica nanospheres as templates, demonstrating that this controllable synthesis method can effectively produce core-shell structured nanoparticles over a wide range of particle sizes. The template method proposed in this paper can significantly improve morphological regularity and size uniformity while effectively reducing the preparation cost of core-shell structured nanoparticles.

## 1. Introduction

Photonic crystals, characterized by their periodic structures at the optical scale, have the capability to regulate the transmission of electromagnetic waves [1,2]. Based on Bragg’s diffraction law [3], the periodic arrangement of nanoparticles of varying sizes can control the propagation of electromagnetic waves across different bands. When the size of the nanoparticles is comparable to the wavelength of visible light, structural colors within the visible range can be produced [4,5]. The diameter [6], refractive index [7], and uniformity [8] of nanoparticles are crucial factors in determining the spectral regulation capability of photonic crystals. Based on their structure, nanoparticles can be classified into single-core and core-shell nanoparticles [9]. Single-core nanoparticles have various preparation methods that are straightforward, but their applications are limited to specific scenarios and functionalization aspects. Core-shell nanoparticles can fully utilize the physical and chemical properties of different compounds [10] to compensate for the deficiencies of single-component compounds in terms of refractive index, stability, and dispersibility. They can also modify surface electrical [11] and magnetic [12] properties to achieve superior electrical or magnetic responsiveness, making them a significant research focus in the field of nanoparticles [13,14,15,16].

To produce structural colors within the visible spectrum, the diameter of the nanoparticles must exceed one hundred nanometers [17], and a high refractive index is also crucial [6]. Ferroferric oxide nanoparticles, known for their high refractive index and superparamagnetic properties, have gained significant attention in the field of photonic crystals. The primary methods for preparing ferroferric oxide nanoparticles include hydrothermal synthesis [18], the sol–gel [19] method, and co-precipitation [20]. Of these methods, ferroferric oxide nanoparticles prepared via hydrothermal synthesis typically have small particle sizes (ranging from tens to several tens of nanometers), exhibit uniform size distribution, do not require high-temperature calcination pretreatment, and can incorporate multivalent ion doping. However, due to the requirement for high-temperature and high-pressure-resistant equipment in the hydrothermal method, the cost is relatively high, making large-scale production challenging. Additionally, producing particles with diameters reaching one hundred nanometers presents issues of uneven particle size and morphological distortions. The sol–gel method can produce high-purity, highly uniform, and large-sized particles, often utilized for preparing particles with complex structures and multilayer shell structures. However, the preparation process is relatively complex, requiring control of multiple process parameters, and it has a long preparation cycle. The co-precipitation method is straightforward and practical, capable of effectively controlling particle size and shape, and it is suitable for large-scale production. However, the particles produced are relatively small, generally ranging from a few nanometers to tens of nanometers, and producing particles with diameters reaching 100 nm is challenging.

**Figure 1:**
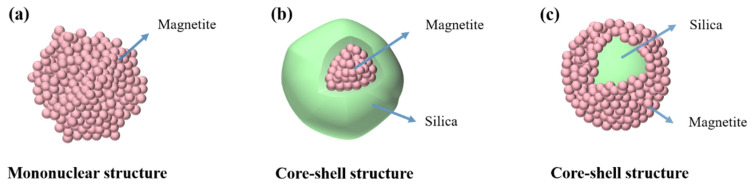
Nanoparticle Models: (**a**) Fe_3_O_4_ nanoparticles, (**b**) Fe_3_O_4_@SiO_2_ nanoparticles, (**c**) SiO_2_@Fe_3_O_4_ nanoparticles.

The preparation cycle of ferroferric oxide nanoparticles is long, and their morphology is difficult to control, especially for large-sized ferroferric oxide (above 100 nm), making it harder to achieve uniform and controllable morphology (Figure 1a). To ensure the uniformity of nanoparticle morphology, researchers coated ferroferric oxide with silica to obtain core-shell structured Fe_3_O_4_@SiO_2_ nanoparticles [21] (Figure 1b). These core-shell structured nanoparticles have shown promising results in the preparation of photonic crystals within the visible wavelength range. However, the method for preparing ferroferric oxide involves small-diameter ferroferric oxide particles clustering into ferroferric oxide nanoparticles within a dispersion system. During the clustering process, various factors such as temperature, reaction time, and the type of dispersant affect nucleation efficiency and the nucleation effect [22,23], resulting in poorer uniformity for larger ferroferric oxide nanoparticles. Consequently, ensuring the uniformity of the core-shell structured nanoparticles obtained by coating with silica is also challenging.

Compared to the high refractive index of ferroferric oxide, silica nanoparticles have mature preparation techniques, simple methods, controllable sizes, and high uniformity [24]; however, their refractive index is low. Therefore, this paper proposes using silica nanoparticles as a template and coating them with Fe_3_O_4_ on their surface to compensate for the low refractive index and significant incoherent scattering of silica. Simultaneously, silica nanoparticles as a template help improve the uniformity of core-shell structured nanoparticles (Figure 1c). Compared with traditional hydrothermal synthesis, the sol–gel method, and the co-precipitation method, the template method has the characteristics of high efficiency, low cost, simple process, and low equipment requirements and has significant advantages in preparing core-shell structured nanoparticles. The effects of factors such as feed amount, modifiers, temperature, and feed order on the coating effect and optimization schemes were explored. The optimized preparation scheme can ensure the uniformity and morphological regularity of the core-shell structured nanoparticles, improving preparation efficiency and reducing experimental requirements. Significant structural colors can be obtained through centrifugal self-assembly. The core-shell structured nanoparticles prepared by the template method possess the physical and chemical properties of different materials and may play an important role as multifunctional composite materials in fields such as secure communication and anti-reaction preparation [25,26].

## 2. Experimental Methods

**Figure 2:**
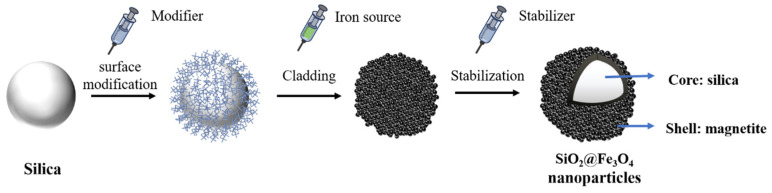
Schematic of template method process for the preparation of SiO_2_@Fe_3_O_4_ core-shell nanoparticles.

Utilizing the enhanced Stöber method, spherical SiO_2_ nanoparticles of various sizes can be synthesized [27]. This technique yields nanoparticles with precisely controllable sizes, high uniformity, and excellent dispersibility, establishing it as the predominant method for nanoparticle synthesis. In this study, 100 nm silica nanoparticles were chosen as the template. To enhance their adsorption capacity for ferroferric oxide (Fe_3_O_4_), HCl (Sinopharm Chemical Reagent Co., Ltd., Shanghai, China), polyethylenimine (PEI) (Sinopharm Chemical Reagent Co., Ltd., Shanghai, China), and NaOH (Aladdin, Shanghai, China) were used as surface modifiers for silica. FeCl_3_·6H_2_O (Sinopharm Chemical Reagent Co., Ltd., Shanghai, China) and FeSO_4_·7H_2_O (Sinopharm Chemical Reagent Co., Ltd., Shanghai, China) supplied the necessary Fe^3+^ and Fe^2+^ ions for synthesis, while ammonia acted as the precipitant and sodium citrate as the stabilizer, enabling the synthesis of SiO_2_@Fe_3_O_4_ core-shell nanoparticles. The effects of the type and concentration of surface modifiers on the adsorption capacity of silica during the Fe_3_O_4_ coating process were investigated (Figure 2), along with the impact of precursor solution dosage, feeding order, and reaction time on the silica coating. Additionally, the influence of external factors such as reaction temperature, stirring speed, ultrasonication, and the presence of sodium citrate on the coating was examined.

### 2.1. Surface Modification of Silica

The surface of silica microspheres carries a negative charge, enabling them to attract positively charged nanoparticles. However, during the initial stages of ferroferric oxide formation, these nanoparticles are not effectively attracted due to weak interactions, resulting in an uneven ferroferric oxide layer on the surface. Therefore, surface modification and functionalization of silica are necessary. Introducing functional groups onto the silica surface increases the binding energy, thereby enhancing the attraction efficiency and binding strength of ferroferric oxide. Consequently, the study employed commonly used acids, bases, and PEI for surface modification.

#### 2.1.1. The Effect of Modifier Types on the Coating of Ferroferric Oxide

Solutions of hydrochloric acid at concentrations of 0.5 mol/L, 1 mol/L, and 1.5 mol/L were prepared. SEM analysis revealed that hydrochloric acid was ineffective for surface modification of silica, and the concentration had no positive effect on the modification outcomes. NaOH was also tested for silica modification, but silica reacts with NaOH, leading to its dissolution. This reaction makes the modification conditions stringent and easily alters the morphology and size of the silica, resulting in poor experimental outcomes. PEI, an excellent surface modifier, is used for various types of particle surface modifications. Experiments revealed that PEI-modified silica could adsorb free ferroferric oxide nanoparticles in the dispersion system, allowing them to grow on its surface and uniformly coat the silica to form a core-shell structure.

**Figure 3:**
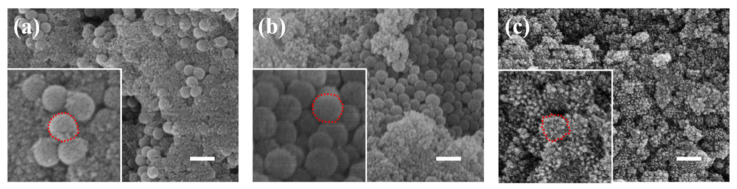
The scanning electron microscope (SEM) images of ferroferric oxide encapsulation after modification with different agents: (**a**) No surface modification, (**b**) surface modification with hydrochloric acid, (**c**) surface modification with PEI (scale bar: 200 nm).

Figure 3 presents the scanning electron microscope (SEM) images of ferroferric oxide encapsulation after modification with different agents. Figure 3a depicts ferroferric oxide on the unmodified silica surface, where the nanoparticles nucleate separately and cluster together without being effectively attracted by the silica microspheres. The illustration in Figure 3a is an enlarged image, with the outline of the nanoparticles circled in red dashed lines. Figure 3b illustrates the surface of silica modified with hydrochloric acid encapsulating ferroferric oxide, showing similar results to the unmodified case, as the ferroferric oxide nanoparticles are still not attracted by the silica. The illustration in Figure 3b is an enlarged image, with the outline of the nanoparticles circled in red dashed lines. Figure 3c demonstrates PEI-modified silica encapsulating ferroferric oxide on its surface, with effective encapsulation, although specific parameters still require optimization. The illustration in Figure 3c is an enlarged image, with the outline of the nanoparticles circled in red dashed lines. The enlarged image shows that small granular crystals form on the surface of silica, and the silica modified with PEI effectively attracts iron oxide. Among the three modifiers, PEI exhibited significant effectiveness. Therefore, this study selected PEI as the surface modifier for silica to attract ferroferric oxide.

#### 2.1.2. The Effect of Modifier Concentration on Nanoparticles

**Figure 4:**
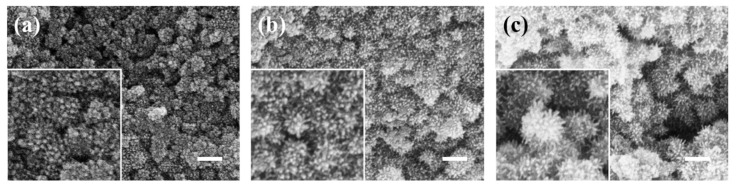
(**a**) Surface modification of silica with 1 mg/mL PEI aqueous solution; (**b**) surface modification of silica with 2 mg/mL PEI aqueous solution; (**c**) surface modification of silica with 4 mg/mL PEI aqueous solution (scale bar: 200 nm).

To determine the optimal PEI modification effect, PEI aqueous solutions of 1 mg/mL, 2 mg/mL, and 4 mg/mL were prepared to modify the surface of silica and characterized by experiments and SEM images. As shown in Figure 4, silica modified with different concentrations of PEI aqueous solutions demonstrated effective attraction to ferroferric oxide. As the concentration of the modifier increased, more functional groups were attached to the silica surface, enhancing its attraction to ferroferric oxide. At high concentrations, the ferroferric oxide on the silica surface accumulates into a sea urchin-like shape. Although it is tightly coated, it no longer maintains a spherical morphology, as shown in Figure 4b,c. The enlarged images in Figure 4b,c show that the sea urchin-shaped spikes formed on the surface of silica become increasingly prominent with the increase of PEI concentration, and there is a tendency for mutual attraction to form larger clusters. Therefore, to achieve a spherical coating, a 1 mg/mL PEI aqueous solution should be used for the surface modification of silica microspheres. Subsequent experiments were conducted using SiO_2_ microspheres modified with a 1 mg/mL PEI solution.

### 2.2. The Effect of Experimental Conditions

During the entire preparation process, the experimental conditions critically impact the product. Firstly, this paper conducts experiments and analyses the effects of reaction temperature, reaction time, mechanical stirring speed, and ultrasonication during the preparation process.

#### 2.2.1. The Effect of Reaction Temperature

**Figure 5:**
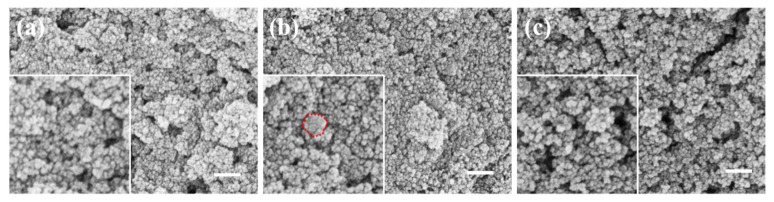
Coating effects at different temperatures: (**a**) 40 °C reaction condition; (**b**) 60 °C reaction condition; (**c**) 80 °C reaction condition (scale bar: 200 nm).

The synthesis process of ferroferric oxide involves coprecipitation, where temperature changes during the reaction affect the rate of product formation. Ferroferric oxide was coated onto the silica surface at temperatures of 40 °C, 60 °C, and 80 °C, respectively. As shown in Figure 5, Figure 5a displays the product synthesized at 40 °C, where a large amount of ferroferric oxide is produced, but the coating effect is poor. Figure 5b shows that at 60 °C, small particles are formed in the dispersion system and uniformly wrapped around the surface of silica (indicated by the red dashed line in the illustration). At 80 °C, as shown in Figure 5c, the formation rate of ferroferric oxide increases, causing small ferroferric oxide nanoparticles to aggregate into larger ones. Considering the quality requirements of the reaction product, 60 °C is a more suitable option.

#### 2.2.2. The Effect of Reaction Time

**Figure 6:**
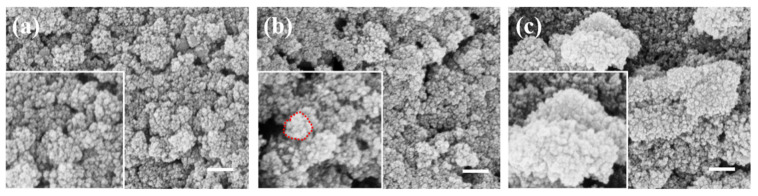
SEM images depicting the coating effects at different reaction times under alkaline conditions: (**a**) Coating status at 10 min of reaction time, (**b**) coating status at 20 min of reaction time, (**c**) coating status at 50 min of reaction time (scale bar: 200 nm).

Appropriate reaction time is crucial for ensuring the progress of the reaction. In this study, reaction times of 10 min, 20 min, and 50 min under alkaline conditions were investigated, as shown in Figure 6. Figure 6a illustrates the coating trend of ferroferric oxide on silica after 10 min of reaction time. Many small particles are generated in a dispersed system, forming a partial coating pattern on the surface of silica. As the reaction time increases, ferroferric oxide continues to be generated and adsorbed on the surface of silica (Figure 6b). However, uneven coating may occur. Further increasing the reaction time leads to a larger amount of ferroferric oxide clustering, forming core-shell structured nanoparticles that aggregate to form larger, indivisible aggregates (Figure 6c). At this point, the coating of ferroferric oxide becomes meaningless. The results suggest that a reaction time of 20 min is optimal, resulting in the most pronounced coating effect and achieving complete particle coating. However, the coating effect is slightly inferior, indicating the need for further exploration of the influence of additional environmental parameters on the coating effect.

#### 2.2.3. The Effect of Mechanical Stirring Speed

**Figure 7:**
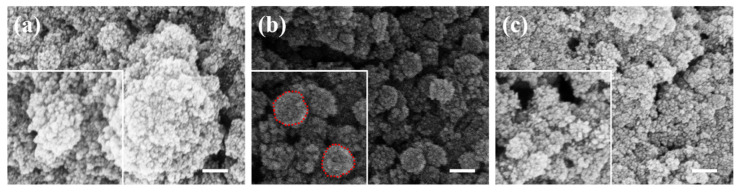
The influence of mechanical stirring speed on the coating effect: (**a**) SEM image of coating at a mechanical stirring speed of 500 rpm; (**b**) SEM image of coating at a mechanical stirring speed of 1000 rpm; (**c**) SEM image of coating at a mechanical stirring speed of 1500 rpm (scale bar: 200 nm).

To ensure the stable occurrence of the reaction, it is indispensable for the reactants to be stably distributed in the dispersion system. The controllable preparation of reaction products depends on the thorough mixing and uniform distribution of reactants during the reaction process. Due to the necessity of secondary feeding and the rapid reaction kinetics involved in the synthesis and growth of ferroferric oxide, high-speed mechanical stirring is employed in such reactions to ensure smooth reaction occurrence. Experiments depicted in Figure 7 were conducted under mechanical stirring at various speeds. In Figure 7a, the SEM image at 500 rpm depicts the reaction’s rapid progression, resulting in a slightly slower dispersion process in the reaction vessel after the addition of ammonia. This leads to the aggregation of generated ferroferric oxide, forming clusters of varying sizes (As shown in the illustration of Figure 7a). Figure 7b illustrates the coating of ferroferric oxide on silica when the mechanical stirrer is set to 1000 rpm. From the SEM image, it is evident that at this speed, the system is relatively uniformly dispersed, and the coating effect is satisfactory. Formed a relatively complete and regular spherical morphology (as shown in the illustration of Figure 7b, the outline of the nanoparticles is drawn by the red dashed line). At 1500 rpm, depicted in Figure 7c, the dispersion system rotates at high speed, resulting in a notable reduction in the velocity of ferroferric oxide clusters and a weakening of silica’s attraction to ferroferric oxide. Despite being chemically synthesized, the coating of ferroferric oxide on silica was inadequate. Subsequent preparations employed mechanical stirring at 1000 rpm to ensure the stability of the synthesis process.

#### 2.2.4. The Impact of Dispersion Methods

**Figure 8:**
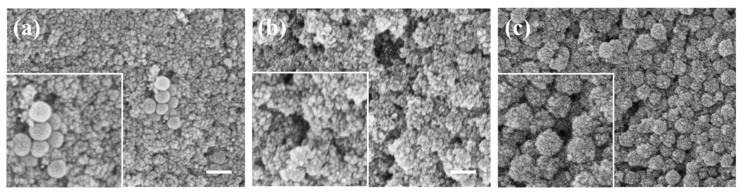
Products obtained by different dispersion methods: (**a**) Product obtained by mechanical stirring dispersion method; (**b**) product obtained by ultrasonic dispersion method; (**c**) product obtained by simultaneous ultrasonic and mechanical stirring dispersion method (scale bar: 200 nm).

Throughout the chemical synthesis process, the uniformity of the dispersion system is disrupted upon the onset of the reaction. To maintain uniformity in the dispersion system throughout the process, ultrasonication is employed in addition to mechanical stirring. Figure 8 depicts the coating of ferroferric oxide on silica using various dispersion methods. The ferroferric oxide coated on silica obtained using the mechanical stirring method in this study, as depicted in Figure 8a, demonstrates relatively uniform dispersion in the system with a good coating effect. To improve the selection of the dispersion method and ensure coating uniformity, ultrasonication was employed in this study to achieve stable dispersion of the system. The results, depicted in Figure 8b, indicate that using ultrasonication for ferroferric oxide coating on silica resulted in a poor coating effect, with significant aggregation of ferroferric oxide. Thus, ultrasonication alone is not suitable for ensuring the stability of the dispersion system. Furthermore, this study combined mechanical stirring with ultrasonication for dispersion of the system, as illustrated in Figure 8c, to enhance the coating effect. Under the dual assurance of mechanical stirring and ultrasonication, the coating effect of ferroferric oxide on silica was significantly improved compared to using only mechanical stirring, resulting in core-shell structured nanoparticles with good coating and relatively uniform size. The results indicate that ultrasound can supplement the insufficiency of mechanical stirring in preparing a uniformly dispersed system and also reduce unnecessary aggregation.

### 2.3. The Impact of Reactants

The primary focus during the experimental reaction process is the reactants themselves. Consequently, this study analyzed the effects of the feeding sequence, stabilizer, stabilizer-catalyst sequence, and iron source ratio on the morphology of the synthesized product.

#### 2.3.1. Feeding Process

During the forward feeding process. 0.5 g of silica microspheres, 0.0811 g of ferric chloride, and 0.0695 g of ferrous sulfate were dispersed in 50 mL of deionized water. The mixture was stirred at 1000 rpm for 15 min until the silica microspheres were evenly dispersed. Subsequently, 1 mL of ammonia solution was added and stirred for an additional 20 min after ferric chloride and ferrous sulfate completely dissolved. Following that, 0.5 mL of sodium citrate solution was added and stirred for 10 min. Following the reaction, magnetic separation was conducted, succeeded by ultrasonic cleaning with deionized water. Centrifugation was then repeated three times to eliminate any remaining unreacted solution and uncoated clusters of ferroferric oxide.

For the reverse feeding process. 0.0811 g of ferric chloride and 0.0695 g of ferrous sulfate were dispersed in 10 mL of deionized water to create solution A. Simultaneously, 0.5 g of silica microspheres were dispersed in 40 mL of deionized water and stirred at 1000 rpm for 15 min. Once the silica microspheres were evenly dispersed, 1 mL of ammonia solution was added and stirred for 1 min. Subsequently, solution A was rapidly added and stirred for 20 min. Following that, 0.5 mL of sodium citrate solution was added and stirred for 10 min. Following the reaction, magnetic separation was conducted, succeeded by ultrasonic cleaning with deionized water. Centrifugation was then repeated three times to eliminate any remaining unreacted solution and uncoated clusters of ferroferric oxide.

**Figure 9:**
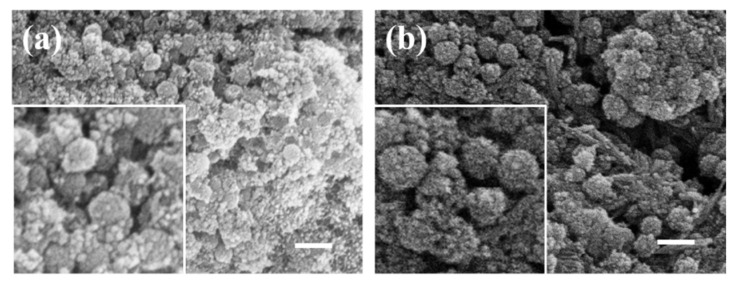
The impact of feeding sequence on the coating effect: (**a**) SEM image of the coating effect with the forward feeding sequence; (**b**) SEM image of the coating effect with the reverse feeding sequence (scale bar: 200 nm).

Figure 9a illustrates the forward feeding sequence, wherein the catalyst (ammonia) is introduced into a dispersion system containing a uniformly distributed iron source and silica. The reaction is intense; consequently, upon the addition of the catalyst, uneven coating may occur due to differences in the local concentration of ammonia, resulting in varying degrees of reaction intensity. Figure 9b depicts the reverse feeding sequence, where the catalyst is introduced into a dispersion system containing uniformly dispersed silica, thus placing the system in an alkaline state prepared for the reaction. Upon the addition of the iron source, despite variations in local concentration, similar reaction rates occur in each part, resulting in a uniform coating. Ammonia, serving as a precipitant, plays a dominant role in determining the reaction rate.

#### 2.3.2. The Effect of Stabilizer

**Figure 10:**
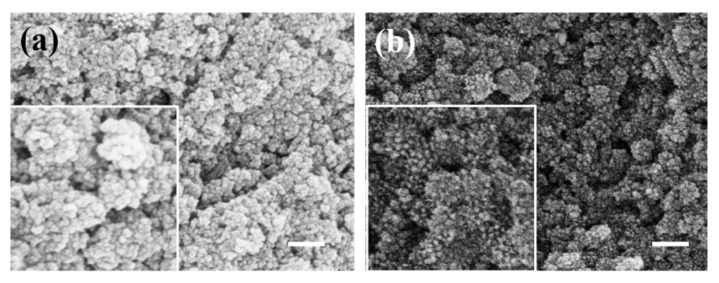
The impact of stabilizer on coating: (**a**) without stabilizer; (**b**) with stabilizer (scale bar: 200 nm).

Sodium citrate, a commonly used stabilizer, is widely employed in nanoparticle synthesis. The addition of stabilizer slows down the reaction, ensuring that the particles maintain chemical equilibrium during growth. Figure 10 illustrates the impact of the stabilizer on the coating process. Figure 10a depicts an SEM image captured during the coating process without the addition of a chemical stabilizer. It shows phenomena such as excessive coating and clustering, which are unfavorable for the generation of core-shell structured nanoparticles. Figure 10b illustrates the coating effect achieved after the addition of stabilizer. The effect of the stabilizer is evident in suppressing the transitional generation and large-scale aggregation of iron oxide, thereby enhancing the coating effect and facilitating the formation of core-shell structured nanoparticles.

#### 2.3.3. Catalyst and Stabilizer Sequence

**Figure 11:**
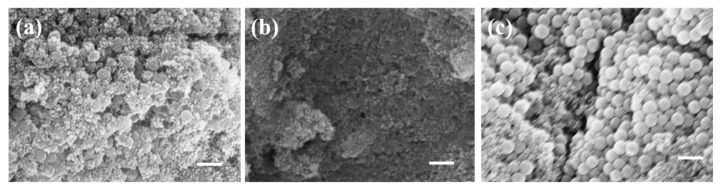
The impact of the addition sequence of catalyst and stabilizer on coating: (**a**) Catalyst first, then stabilizer; (**b**) stabilizer first, then catalyst; (**c**) simultaneous addition of stabilizer and catalyst (scale bar: 200 nm).

Catalysts are crucial reagents that drive reactions, while stabilizers ensure the morphology of the final product; together, they complement each other. To investigate their effects on coating, this study conducted comparative experiments on the sequence of adding both catalyst and stabilizer. Figure 11 illustrates the effects of different sequences of adding catalyst and stabilizer on the coating process. Figure 11a presents SEM images of adding the catalyst first and then the stabilizer. The addition of a catalyst triggers the reaction, and after some time, the stabilizer intervenes to stabilize the generation and coating of iron oxide, suppressing adverse changes in the core-shell nanostructure and ensuring its integrity. Figure 11b shows the effect of adding stabilizer first, followed by the addition of catalyst. The addition of a stabilizer inhibits the reaction process, resulting in the generation of small-sized iron oxide nanoparticles floating on the surface. Figure 11c demonstrates the effect of adding both catalyst and stabilizer simultaneously. Their simultaneous action leads to the neutralization of acidity, weakening alkaline catalysis, and resulting in the unsatisfactory synthesis of iron oxide, with hardly any occurrence of coating phenomenon.

#### 2.3.4. Iron Source Ratio

**Figure 12:**
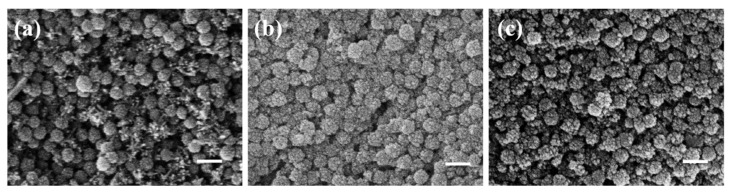
The effect of iron concentration on coating performance: (**a**) Coating effect with iron concentration at 0.5 times; (**b**) coating effect with iron concentration at 1 time; (**c**) coating effect with iron concentration at 2 times (scale bar: 200 nm).

To ensure the synthesis of ferroferric oxide, the molar ratio of Fe^3+^ provided by ferric chloride and Fe^2+^ provided by ferrous sulfate in the solution is consistently maintained at 2:1. The coating effect was observed by adjusting the proportions of ferric chloride and ferrous sulfate in the solution. Figure 12a demonstrates that reducing the feed amount to 50% of the initial amount results in uneven or uncoated phenomena, as observed by SEM imaging. Conversely, increasing the feed amount to 200% of the initial amount, as depicted in Figure 12c, leads to excessive or leaked coating phenomena. This occurs due to the presence of large amounts of free Fe^3+^ and Fe^2+^ ions in the solution. After the addition of ammonia, a significant number of nanoscale ferroferric oxide particles rapidly nucleate. Some particles are adsorbed onto the surface of silicon dioxide and continue to grow, while others aggregate to form new nuclei. When the amount of iron source is 100%, as depicted in Figure 12b, ferroferric oxide can be uniformly coated on the surface of silicon dioxide, forming core-shell structured nanoparticles.

## 3. Results and Discussion

### 3.1. SiO_2_@Fe_3_O_4_ Characterization of Core-Shell Structured Nanoparticles

**Figure 13:**
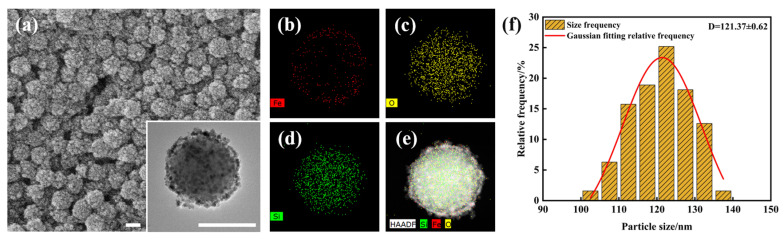
The morphology and elemental distribution of SiO_2_@Fe_3_O_4_ nanoparticles: (**a**) SEM image of SiO_2_@Fe_3_O_4_ nanoparticles (inset: TEM image of a single nanoparticle); (**b**) distribution map of iron elements in SiO_2_@Fe_3_O_4_ nanoparticles; (**c**) distribution map of oxygen elements in SiO_2_@Fe_3_O_4_ nanoparticles; (**d**) distribution map of silicon elements in SiO_2_@Fe_3_O_4_ nanoparticles; (**e**) distribution map of the fusion of iron, oxygen, and silicon elements in SiO_2_@Fe_3_O_4_ nanoparticles (scale bar: 100 nm); (**f**) size distribution curve of SiO_2_@Fe_3_O_4_ nanoparticles.

The nanocomposite particles prepared using the optimized final conditions are characterized, as depicted in Figure 13. Figure 13a displays the SEM image of SiO_2_@Fe_3_O_4_ nanoparticles, demonstrating a good encapsulation effect of ferroferric oxide on silica without any omissions observed. As depicted in the inset of Figure 13a, the TEM results of the nanoparticles prepared in this study reveal a clear core-shell structure, with the shell composed of clusters of small-sized ferroferric oxide nanoparticles, resulting in a uniform and complete encapsulation. Figure 13b illustrates the distribution of iron elements in the core-shell nanoparticles. Due to the presence of iron source components in the shell, the distribution of iron is only observed in the shell, consistent with the TEM imaging results. Figure 13c depicts the distribution of oxygen elements. As the core-shell consists of silica and ferroferric oxide, respectively, the oxygen elements are evenly distributed in both the core and shell, akin to the distribution range of iron elements. Figure 13d illustrates the distribution of silicon elements, with a distribution range significantly smaller than that of iron and oxygen elements. Figure 13e illustrates the combined distribution of iron, oxygen, and silicon elements. This distribution confirms the composition of the core-shell structured nanoparticles, with silica as the core and ferroferric oxide as the shell. The size distribution curve of SiO_2_@Fe_3_O_4_ is shown in Figure 13f, with an average particle size of 121.37 nm.

**Figure 14:**
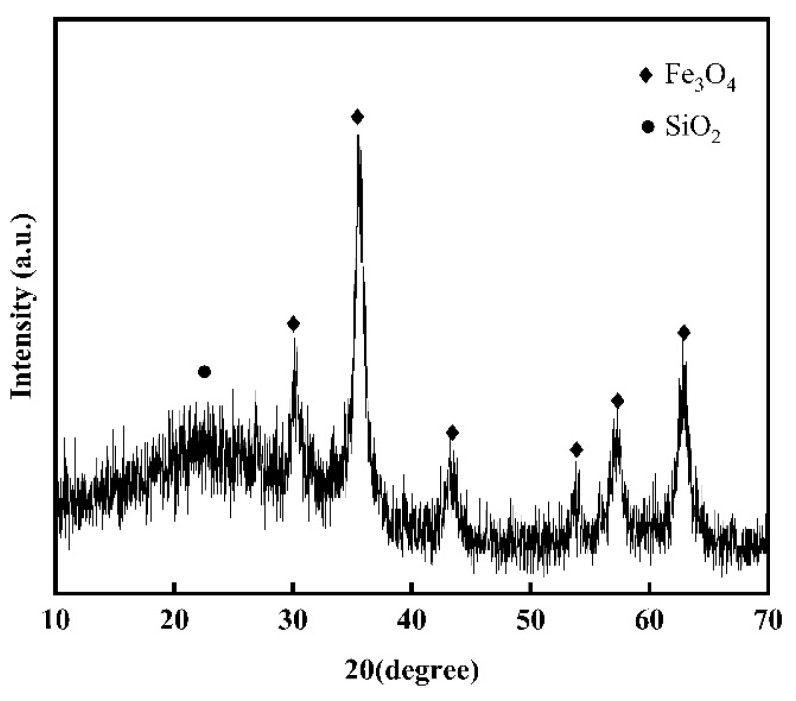
The X-ray diffraction pattern of SiO_2_@Fe_3_O_4_ core-shell structured nanoparticles.

X-ray diffraction (XRD) analysis was conducted on the core-shell structured nanoparticles prepared in this study, as depicted in Figure 14, over a 2θ angle range of 10° to 70°. A diffraction peak of silicon dioxide is observed at 22.7°, characterized by a broad half-peak width, indicative of the amorphous structure of the silicon dioxide. Diffraction peaks are observed at 30.3°, 35.7°, 43.3°, 53.9°, 57.3°, and 62.9°, corresponding to the diffraction of Fe_3_O_4_ crystal planes (220), (311), (400), (422), (511), and (440), respectively. These peaks match the characteristic peaks in the XRD pattern of Fe_3_O_4_ provided by the International Center for Diffraction Data (PDF No. 65-3107), with their sharpness indicating the good crystallinity of ferroferric oxide.

The crystal grain size *d* of ferroferric oxide can be calculated from its diffraction peaks using the Debye–Scherrer equation [28].

(1) d=kλ/(βcosθ)

In this equation, *d* represents the crystal grain size in nanometers (nm); *λ* is the wavelength of the diffracted light, typically taken as 0.15046 nm; *β* is the full width at half maximum (FWHM) in degrees; *θ* is half the diffraction angle in degrees; *k* is a constant, usually taken as 0.89. By calculation, the crystal grain size of ferroferric oxide is determined to be 13.60 nm, indicating that the shell layer on the surface of silicon dioxide is formed by clusters of small-sized ferroferric oxide nanoparticles.

**Figure 15:**
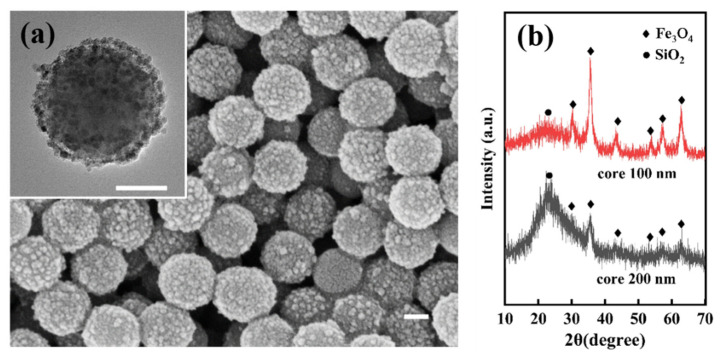
Characterization of SiO_2_ surface wrapped with Fe_3_O_4_ particles with a size of 200 nm: (**a**) SEM image of SiO_2_@Fe_3_O_4_ core-shell nanoparticles (TEM image in the inset) (scale bar: 100 nm); (**b**) XRD pattern of SiO_2_ wrapped with Fe_3_O_4_ of different sizes.

The results of coating iron oxide on the surface of 200 nm silica nanoparticles are depicted in Figure 15. Figure 15a displays the SEM image of iron oxide coating around 200 nm silica, exhibiting uniform coating, a well-defined spherical morphology, and an absence of omissions. The inset presents its TEM image, revealing a clear core-shell structure with evenly distributed iron oxide in the shell. Figure 15b presents a comparison of the XRD patterns of SiO_2_@Fe_3_O_4_ core-shell nanoparticles of two sizes. The characteristic peaks of silica and iron oxide align at the same positions, with no other interference peaks or deviations, affirming the purity of both core-shell nanoparticles. Conversely, the peak height and area of silica in nanoparticles with a 200 nm core are significantly higher than those in nanoparticles with a 100 nm core, suggesting that the silica content in the 200 nm core nanoparticles is markedly higher than that in the 100 nm core nanoparticles. This result is consistent with the experiment itself. Meanwhile, the experiment proves that for cores of different sizes, perfect coating of silica by ferroferric oxide can be achieved. The method is simple, has a short preparation time, low equipment requirements, and high efficiency. Two different sizes of core-shell structured nanoparticles prepared by the template method have stable structures and no significant changes after being stored for 3 months under laboratory conditions.

### 3.2. Preparation of Visible Light Photonic Crystals

**Figure 16:**
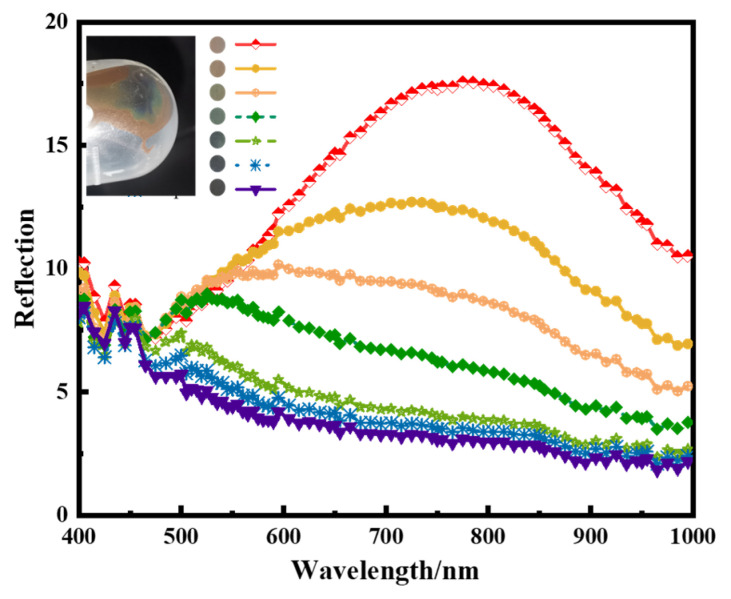
Reflection spectrum curve of centrifugally self-assembled photonic crystal (insertion is the physical picture of the photonic crystal).

SiO_2_@Fe_3_O_4_ core-shell nanoparticles, featuring a 100 nm core size, demonstrate centrifugal self-assembly, yielding reflection rainbow-colored photonic crystals. These crystals reflect wavelengths spanning the visible light spectrum. Figure 16 illustrates spectral curves corresponding to various reflection wavelengths ranging from 775 nm to 455 nm at different positions, providing a wavelength coverage range of 320 nm.

## 4. Conclusions

This paper prepared SiO_2_@Fe_3_O_4_ core-shell structured nanoparticles and explored the conditions for silica modification and the influencing factors in the preparation of ferroferric oxide, including temperature, catalysts, stabilizers, stirring speed, and feed amount on the coating effect. The results indicate that at a stirring speed of 1000 rpm, a temperature of 60 °C, and under ultrasonic conditions, the prepared ferroferric oxide can achieve a perfect coating of silica nanoparticles of various sizes modified with agents, resulting in stable and uniformly sized core-shell structured nanoparticles. The study also demonstrated that the self-assembly of SiO_2_@Fe_3_O_4_ core-shell structured nanoparticles of specific sizes can yield rainbow-colored photonic crystals within the visible spectrum. This type of photonic crystal can be used in fields such as optical switching and band filtering and also has potential value in secure communication and anti-counterfeiting. Core-shell structured nanoparticles of different sizes can be used to prepare photonic crystal devices for various wavelength bands. These findings provide valuable references for nanoparticle synthesis, preparation, and exploration of influencing factors.

## 5. Optimized Preparation Steps

### 5.1. Optimal Preparation Conditions

SiO_2_ concentration for modification is 5 mg/mL, PEI is used as a modifier with a concentration of 1 mg/mL, and the modification time is 30 min. During the coating process, a reverse feeding sequence is adopted, with a SiO_2_ concentration of 1 mg/mL, Fe^3+^ concentration of 0.01 mmol/mL, Fe^2+^ concentration of 0.005 mmol/mL, and coating time of 20 min. Sodium citrate is used as a stabilizer with an addition amount of 0.1 mg/mL and a stabilization time of 10 min. The reaction temperature is 60 °C, and the reaction process is carried out under the combined action of mechanical stirring at 1000 rpm and ultrasound.

### 5.2. Surface Modification Steps of Silica

Initially, 0.1 g of PEI was dissolved in 100 mL of deionized water to produce a 1 mg/mL PEI dispersion. Subsequently, 0.5 g of SiO_2_ was added to the dispersion and sonicated for 30 min. The resulting mixture was washed twice with deionized water, dried, and ground into powder for later use.

### 5.3. Preparation of SiO_2_@Fe_3_O_4_

Firstly, 0.0811 g of FeCl_3_·6H_2_O and 0.0695 g of FeSO_4_·7H_2_O were separately dissolved in 10 mL of deionized water and sonicated for 15 min to form a brown homogeneous dispersion labeled as A. Then, 0.1 g of sodium citrate was dissolved in 10 mL of deionized water to form solution B. 0.05 g of modified SiO_2_ was dissolved in 40 mL of deionized water to form solution C, stirred with a magnetic stirrer for 15 min, then 1 mL of ammonia was added after SiO_2_ was uniformly dispersed in deionized water, and stirred for 1 min. After the ammonia was uniformly dispersed, solution A was added, and the solution quickly turned black. The temperature was maintained at 60 °C, stirred with a magnetic stirrer for 20 min, then 0.5 mL of solution B was added and stirred for another 10 min. The resulting black product was collected with a strong magnet, washed three times with deionized water under ultrasonication, and dissolved in an appropriate amount of deionized water for later use. The entire preparation process was conducted under sonication, with a stirring speed of 1000 rpm and a reaction temperature of 60 °C.

## Data Availability

The original contributions presented in the study are included in the article, further inquiries can be directed to the corresponding author.

## References

[1] Liang Y., Liang Z., Liu Z., Jun P., Qiu D. (2023). Study on the transmission characteristics and band structure of 2D and 3D plasma photonic crystals. Opt. Express.

[2] Lv X., Zhong B., Huang Y., Xing Z., Wang H., Guo W., Zhang Z. (2023). Research progress in preparation and application of photonic crystals. Chin. J. Mech. Eng..

[3] Inoue T., Morita R., Nigo K., Yoshida M., De Zoysa M., Ishizaki K., Noda S. (2023). Self-evolving photonic crystals for ultrafast photonics. Nat. Commun..

[4] Liu J., Nero M., Jansson K., Willhammar T., Sipponen M.H. (2023). Photonic crystals with rainbow colors by centrifugation-assisted assembly of colloidal lignin nanoparticles. Nat. Commun..

[5] Jia R., Xiang S., Wang Y., Chen H., Xiao M. (2024). Electrically Triggered Color-Changing Materials: Mechanisms, Performances, and Applications. Adv. Opt. Mater..

[6] Madanu T.L., Mouchet S.R., Deparis O., Liu J., Li Y., Su B.L. (2023). Tuning and transferring slow photons from TiO_2_ photonic crystals to BiVO_4_ nanoparticles for unprecedented visible light photocatalysis. J. Colloid Interface Sci..

[7] Sorathiya V., Lavadiya S., Faragallah O.S., Eid M.M., Rashed A.N.Z. (2022). D shaped dual core photonics crystal based refractive index sensor using graphene–titanium–silver materials for infrared frequency spectrum. Opt. Quantum Electron..

[8] Li H., Wu P., Zhao G., Guo J., Wang C. (2021). Fabrication of industrial-level polymer photonic crystal films at ambient temperature Based on uniform core/shell colloidal particles. J. Colloid Interface Sci..

[9] Nikzamir M., Akbarzadeh A., Panahi Y. (2021). An overview on nanoparticles used in biomedicine and their cytotoxicity. J. Drug Deliv. Sci. Technol..

[10] Ma X., Li Y.Y., Hussain I., Shen R., Yang G., Zhang K. (2020). Core–shell structured nanoenergetic materials: Preparation and fundamental properties. Adv. Mater..

[11] Chen S., Cheng Y., Zhao Z., Zhang K., Hao T., Sui Y., Wang W., Zhao J., Li Y. (2023). Core–Shell-Structured Electrorheological Fluid with a Polarizability-Tunable Nanocarbon Shell for Enhanced Stimuli-Responsive Activity. ACS Appl. Mater. Interfaces.

[12] Wang Z., Valenzuela C., Xue P., Zhang X., Zhang X., Chen Y., Yang Y., Wang L., Xu X. (2022). Magnetic structural color hydrogels for patterned photonic crystals and dynamic camouflage. ACS Appl. Polym. Mater..

[13] Wang D., Li J., Sun X., Hu J., Tan X., Jia Q., Liu J., Zhang X., Wu G., Wang X. (2024). New electric field responsive photonic crystals with remarkable yellow-to-green switch for adaptive camouflage. J. Colloid Interface Sci..

[14] Chen Y., Sun W., Zheng H., Li C., Zhang B., Wang B., Hao C. (2021). The electrorheological response behavior of small coral-like H_2_Ti_2_O_5_@SiO_2_ core-shell nanoparticles. J. Taiwan Inst. Chem. Eng..

[15] Hao L.W., Liu J.D., Li Q., Qing R.K., He Y.Y., Guo J., Li G., Zhu L., Xu C., Chen S. (2021). Microfluidic-directed magnetic controlling supraballs with multi-responsive anisotropic photonic crystal structures. J. Mater. Sci. Technol..

[16] Park J.G., Rogers W.B., Magkiriadou S., Kodger T., Kim S.H., Kim Y.S., Manoharan V.N. (2017). Photonic-crystal hydrogels with a rapidly tunable stop band and high reflectivity across the visible. Opt. Mater. Express.

[17] Cai Z., Li Z., Ravaine S., He M., Song Y., Yin Y., Zheng H., Teng J., Zhang A. (2021). From colloidal particles to photonic crystals: Advances in self-assembly and their emerging applications. Chem. Soc. Rev..

[18] Jesus A.C.B., Jesus J.D., Lima R.J.S., Moura K.O., Almeida J.M.A., Duque J.G.S., Meneses C.T. (2020). Synthesis and magnetic interaction on concentrated Fe_3_O_4_ nanoparticles obtained by the co-precipitation and hydrothermal chemical methods. Ceram. Int..

[19] Imran M., Riaz S., Shah S.M.H., Batool T., Khan H.N., Sabri A.N., Naseem S. (2020). In-vitro hemolytic activity and free radical scavenging by sol-gel synthesized Fe_3_O_4_ stabilized ZrO_2_ nanoparticles. Arab. J. Chem..

[20] Ba-Abbad M.M., Benamour A., Ewis D., Mohammad A.W., Mahmoudi E. (2022). Synthesis of Fe_3_O_4_ nanoparticles with different shapes through a co-precipitation method and their application. JOM.

[21] Xu J., Zhao Q., Hu T., Chen X., Cao Y. (2021). Rapid preparation of size-tunable Fe_3_O_4_@SiO_2_ nanoparticles to construct magnetically responsive photonic crystals. J. Nanopart. Res..

[22] Wei Y., Han B., Hu X., Lin Y., Wang X., Deng X. (2012). Synthesis of Fe_3_O_4_ nanoparticles and their magnetic properties. Procedia Eng..

[23] Kulkarni S.A., Sawadh P.S., Palei P.K., Kokate K.K. (2014). Effect of synthesis route on the structural, optical and magnetic properties of Fe_3_O_4_ nanoparticles. Ceram. Int..

[24] Park S.K., Do Kim K., Kim H.T. (2002). Preparation of silica nanoparticles: Determination of the optimal synthesis conditions for small and uniform particles. Colloids Surf. A.

[25] Lalegani Z., Ebrahimi S.A.S., Hamawandi B., La Spada L., Batili H., Toprak M.S. (2022). Targeted dielectric coating of silver nanoparticles with silica to manipulate optical properties for metasurface applications. Mater. Chem. Phys..

[26] Lincoln R.L., Scarpa F., Ting V.P., Trask R.S. (2019). Multifunctional composites: A metamaterial perspective. Multifunct. Mater..

[27] Ghimire P.P., Jaroniec M. (2021). Renaissance of Stöber method for synthesis of colloidal particles: New developments and opportunities. J. Colloid Interface Sci..

[28] Karthikeyan B., Vettumperumal R. (2022). Structural and optical characterization of Mg_2_SiO_4_ and Mg_2_SiO_4_-Pr_6_O_(11)_ nanocomposite for optical devices. Opt. Mater. Jan..

